# New York Polyclinic Medical School and Hospital

**Published:** 1911-03

**Authors:** John A. Wyeth

**Affiliations:** New York


### New York Polyclinic Medical School and Hospital.

NEW YORK, February 8, 1911.

*Dear Doctor Daniel:*

I am sending you a pamphlet descriptive of the new school and hospital building of the New York Polyclinic.

As an old friend of this institution, I know that you and the 20,000 physicians who have worked with us in the old building in East Thirty-fourth Street will rejoice with me and my colleagues in the fact that we are soon to have one of the largest and best equipped plants for post-graduate instruction in the world.

Two friends of medical education living in New York City have made donations amounting to $375,000, and we are now spending $600,000 upon the main building.

We have been assigned the city ambulance and emergency service for a large area on the west side, in which the new hospital is being built, and will be able to quadruple the number of patients treated, and to double the number of physicians in our classes.

With all good wishes. Ever sincerely yours,

JOHN A. WYETH.

